# Conocimiento de la población general y de familiares de pacientes con epilepsia sobre primeros auxilios ante una crisis epiléptica

**DOI:** 10.23938/ASSN.1147

**Published:** 2026-03-06

**Authors:** Alejandro Viloria-Alebesque, Ángela Bono-Velilla, Elena Bellosta-Diago, María Pilar Navarro-Pérez, Nuria Reyes-Perera, Sonia Santos-Lasaosa

**Affiliations:** 1 Servicio de Neurología Hospital Clínico Universitario Lozano Blesa Zaragoza España; 2 Instituto de Investigación Sanitaria Aragón (IIS) Zaragoza España; 3 Servicio de Endocrinología y Nutrición Hospital General Universitario de Castellón Castellón España; 4 Servicio de Neurología Hospital Obispo Polanco Teruel España

**Keywords:** Epilepsia, Crisis Epiléptica, Primeros Auxilios, Conocimientos, Actitudes y Práctica en Salud, Encuestas, Epilepsy, Seizures, First Aid, Health Knowledge, Attitudes Practice, Surveys and Questionnaires

## Abstract

**Fundamento:**

Las crisis epilépticas suelen ocurrir como episodios paroxísticos y autolimitados, por lo que frecuentemente son presenciadas y atendidas por personas no cualificadas. El objetivo de este estudio es valorar el conocimiento sobre primeros auxilios ante una crisis epiléptica en dos grupos: población general y familiares de pacientes con epilepsia.

**Metodología:**

Se realizó una encuesta estructurada a personas de la población general y a familiares sobre diferentes aspectos de manejo y maniobras a realizar ante una crisis epiléptica. Constaba de 16 preguntas: una de respuesta abierta, ocho con respuesta de opción múltiple, más siete específicas para familiares.

**Resultados:**

El 39,2% de las 434 encuestas fueron respondidas por familiares. Las maniobras adecuadas (permanecer junto al paciente, registrar duración de la crisis, limpiar secreciones) fueron significativamente más frecuentes en familiares y las inadecuadas (abrir la boca con algún objeto, sujetar al paciente) en población general, sin diferencias para la posición del paciente. La fuente de información principal para familiares era el personal sanitario, y las redes sociales y medios de comunicación para la población general. En el grupo de familiares, el 51,1% no daría medicación, pero sí llamarían a emergencias ante determinadas situaciones (62%) o si la crisis duraba más de cinco minutos (54,5%).

**Conclusiones:**

El nivel de conocimiento sobre primeros auxilios ante una crisis epiléptica fue subóptimo en ambos grupos, pero especialmente en la población general. Por tanto, se necesitan estrategias para fomentar y mejorar la formación sanitaria de población general y familiares sobre primeros auxilios ante crisis epilépticas.

## Abstract (en)

**Background:**

Epileptic seizures are typically paroxysmal and self-limiting events that are frequently witnessed and managed by laypersons. This study aimed to assess knowledge of first aid for epileptic seizures in two groups: the general population and relatives of patients with epilepsy.

**Methods:**

A structured survey was administered to members of the general population and to family members of patients with epilepsy. The questionnaire assessed knowledge of appropriate management and first aid maneuvers during a seizure. It included 16 questions: one open-ended item, eight multiple-choice questions, and seven additional questions directed specifically to family members.

**Results:**

Of the 434 respondents, 39.2% were family members of patients with epilepsy. Appropriate measures - such as remaining with the patient, timing the seizure, and cleaning secretions - were reported significantly more often by family members. Inappropriate actions - including placing objects in the patient’s mouth and restraining movements - were more common among the general population. No significant differences were observed regarding patient positioning. Family members most frequently identified healthcare professionals as their primary source of information, whereas the general population cited social media and mass media. Among family members, 51.1% would not administer medication; 62% would call emergency services in specific situations, and 54.5% would do so if the seizure lasted more than five minutes.

**Conclusions:**

Knowledge of seizure first aid was suboptimal in both groups, particularly in the general population. Targeted education strategies are needed to improve first aid training for both relatives and the broader community.

## INTRODUCCIÓN

La crisis epiléptica (CE) se define como un conjunto de signos y síntomas neurológicos de aparición súbita y habitualmente transitoria debido a una descarga neuronal anormal, excesiva y sincrónica en el cerebro^1^. Las CE son por lo tanto paroxísticas, de duración breve (no mayor de 2-3 minutos en la mayoría de los casos^2^), e imprevisibles, por lo que en muchas ocasiones ocurren fuera del ámbito hospitalario, y por lo tanto pueden ser presenciadas y atendidas en un primer momento por personas no cualificadas.

Las CE tienen riesgo de complicaciones asociadas como traumatismos, broncoaspiración, estado epiléptico, accidentes y parada cardiorrespiratoria^3^, por lo que la adopción de unas medidas básicas y una atención adecuada desde un primer momento es imprescindible para evitarlas o, al menos, minimizarlas. Estos pacientes tienen, además, un riesgo añadido de muerte prematura^4^ y mayor probabilidad de padecer muerte súbita (SUDEP)^5^. Por lo tanto, la educación sanitaria respecto a las medidas básicas de atención a una CE, dirigida tanto a familiares y personas en contacto estrecho con pacientes con epilepsia como a la población general, tiene un valor notable. Existen distintos trabajos de investigación que valoran el conocimiento sobre primeros auxilios ante una CE^6^^-^^13^. Sin embargo, muchos de ellos se han realizado en países en vías de desarrollo^6^^,^^8^^,^^10^^,^^11^ y, por otra parte, se han dirigido a colectivos profesionales concretos, como profesores o personal sanitario^7^^-^^10^^,^^12^^,^^13^.

En España, el estudio EPIBERIA mostró una prevalencia global de epilepsia de 14,87 casos por 1.000 personas mayores de 18 años, de los cuales 5,79 casos por 1.000 tenían una epilepsia activa^14^. Teniendo en cuenta estos datos y el carácter paroxístico e imprevisible de las CE, es relevante evaluar el conocimiento de la población sobre primeros auxilios ante una CE. Y lo es más si se tienen en consideración lo ampliamente extendidas que se encuentran ciertas creencias erróneas y peligrosas respecto a las CE^15^, y las experiencias que relatan los propios pacientes tras sufrir una CE en un entorno público sobre la inseguridad y desconocimiento en la forma de actuar de terceras personas^16^, y la baja confianza de la población adulta en sus propias capacidades para atender a una persona que está sufriendo una CE^17^.

Por ello, el objetivo del presente trabajo es evaluar en nuestro medio el nivel del conocimiento sobre primeros auxilios ante una CE, tanto en sujetos de la población general como en familiares de pacientes con epilepsia.

## MATERIAL Y MÉTODOS

Estudio observacional transversal para analizar los conocimientos sobre primeros auxilios ante una CE en personas con y sin contacto con pacientes con epilepsia mediante encuestas realizadas entre febrero de 2022 y diciembre de 2023 en Zaragoza (España).

Se seleccionaron dos grupos de estudio:

- población general, reclutado en el entorno no sanitario de los autores y entre acompañantes de pacientes sin epilepsia de la consulta de neurología general del Hospital General de la Defensa de Zaragoza;
- familiares de pacientes con epilepsia, reclutado entre acompañantes de dichos pacientes que acuden a la consulta de epilepsia del Hospital General de la Defensa y del Hospital Clínico Universitario Lozano Blesa (Zaragoza).

Los criterios de exclusión en ambos grupos fueron ser menor de 18 años y tener formación académica sanitaria.

La encuesta fue diseñada y revisada por los autores atendiendo a las recomendaciones de actuación en primeros auxilios básicos ante una CE del *Documento de Consenso para el tratamiento de CE*^18^ confeccionado entre la Sociedad Española de Neurología (SEN), Sociedad Española de Epilepsia y Sociedad Española de Urgencias y Emergencias, y del *Manual de Práctica Clínica en Epilepsia* de la SEN^19^, abarcando en su diseño la valoración de los principales y diferentes aspectos de la actuación clínica referida. El cuestionario (Anexo I) incluye:

- Datos demográficos: edad, sexo, medio urbano o rural, y nivel de formación académica (estudios superiores o no).
- Preguntas sobre el conocimiento sobre primeros auxilios ante una CE:
  - (1) Pregunta inicial abierta general sobre las acciones que la persona realizaría ante una CE. Se analiza si en dicha respuesta aparecen seis acciones concretas, tanto adecuadas como inadecuadas: colocar a la persona en posición lateral de seguridad, registrar el tiempo de duración de la CE, introducción de algún objeto en la cavidad oral, despejar la vía aérea, aflojar ropa ajustada, y llamar al 061/112.
  - (2-8) Preguntas cerradas sobre diferentes posibles acciones a realizar sobre la persona que sufre la CE, con tres opciones de respuesta (sí/no/no lo sé) de las que únicamente una de ellas se considera respuesta adecuada (RA): permanencia con la persona que sufre una CE (RA: sí), registro del tiempo de duración de la CE (RA: sí); colocación del paciente en alguna posición (cuatro opciones: no cambiar de posición/decúbito lateral/decúbito supino/decúbito prono; RA: decúbito lateral), limpieza de secreciones orales (RA: sí), colocación de un objeto en la cavidad oral (RA: no), sujeción del paciente (RA: no).
  - (9) Pregunta sobre la forma de aprendizaje de los conocimientos sobre el tema con cuatro opciones: formación en centros educativos o cursos/ medios de comunicación y/o redes sociales/ explicaciones por personal sanitario/ otros.
  - (10-16) Preguntas exclusivas para el grupo de familiares. Administración de medicación ante una CE (opciones: sí/no/no lo sé), medicación que administraría (respuesta abierta), prescriptor de la medicación (opciones: médico de atención primaria/ neurólogo/ otros); llamada al 061/112 (opciones: sí, siempre/ no, nunca/ solo en ciertas situaciones); tiempo de duración de la CE a partir del cual llamaría al 061/112 (opciones: 1/ 3/ 5/ 10 minutos); en qué otras situaciones avisaría al 061/112 (respuesta abierta). Finalmente, pertenencia a una asociación de pacientes con epilepsia y/o familiares.

Las personas que cumplían los criterios de inclusión fueron informados por los investigadores sobre el estudio y, si accedían a participar, se les suministraba la encuesta en formato físico, completándola sin recoger datos identificativos y sin disponer de un límite de tiempo para ello (globalmente precisaron en torno a 5-10 minutos).

El protocolo de estudio fue valorado con dictamen favorable por parte del Comité de Ética de la Investigación de la Comunidad Autónoma de Aragón (PI22/151).

### Análisis estadístico

Se realizó un análisis descriptivo de las variables mediante la media y desviación estándar (DE) en las variables cuantitativas, y frecuencias absolutas y porcentajes para las variables categóricas. Posteriormente se compararon mediante las pruebas t-Student y Chi-cuadrado, respectivamente. Se han utilizado modelos de regresión logística multivariante para analizar el efecto de los grupos ajustado por las variables demográficas sobre las respuestas. Los análisis se realizaron mediante el paquete informático SPSS v.21, considerándose resultados estadísticamente significativos aquellos con valor p <0,05 en prueba bilateral.

## RESULTADOS

El estudio recogió 434 encuestas respondidas mayoritariamente por mujeres (67,7%), con media de edad 47,1 años (rango: 18 a 91), residentes en medio urbano (76,7%) y con estudios superiores (59,2%). El 39,8% de las encuestas correspondían a familiares de pacientes con epilepsia y el resto a la población general (60,8%). No se observaron diferencias significativas entre ambos grupos en relación a las variables demográficas.

Respecto a las acciones a realizar ante una CE (pregunta abierta), colocar al paciente en posición lateral de seguridad, registrar el tiempo de duración de la crisis y no introducir objetos en la boca del paciente fueron significativamente más mencionadas por el grupo de familiares, mientras que llamar al 061, introducir objetos en la boca, despejar la vía aérea y aflojar la ropa, estas dos últimas sin diferencias significativas, fueron más frecuentes en el grupo de población general (Tabla 1).

**Tabla 1:** Respuesta a la pregunta abierta inicial del cuestionario: ¿Cómo actuaría si presenciara que una persona sufre una crisis epiléptica/convulsiones?

Acciones	Frecuencia de respuesta (%)			OR (IC95%)
	Total	Población general	Familiares	p (X^2^)
Colocar en decúbito lateral	41,9	35,6	51,8	1,9 (1,3-2,9)
				0,001
Registrar tiempo de duración	7,6	2,7	15,3	6,6 (2,8-15)
				0,001
Llamar al 061	38,7	47,3	25,3	0,4 (0,2-0,6)
				0,001
Despejar vía aérea	3,9	4,2	3,5	0,8 (0,3-2,3)
				0,738
Aflojar la ropa	2,1	3	0,6	0,2 (0,1-1,5)
				0,081
No colocar objetos en la boca	2,3	1,1	4,1	3,7 (1-14)
				0,043
Colocar objetos en la boca	24,9	31,4	14,7	0,4 (0,2-0,6)
				0,001

OR: *odds ratio*; IC: intervalo de confianza.

Respecto a las posibles acciones a realizar sobre la persona que sufre la CE, incluidas como preguntas de opción múltiple, permanecer con la persona que está sufriendo la CE, cronometrar el tiempo de duración del episodio y limpiar las secreciones de la cavidad oral fueron respuestas significativamente más frecuentes en el grupo de familiares, mientras que el grupo de población general realizaría más habitualmente dos acciones consideradas inadecuadas(introducir algún objeto en la boca del paciente y sujetarlo) (Tabla 2). En el análisis multivariante de los ítems que mostraban diferencias significativas entre ambos grupos, ajustado respecto a las variables demográficas, no se observó que las variables demográficas fueran variables independientes predictoras de la respuesta.

**Tabla 2:** Frecuencia [n (%)] de las respuestas a preguntas de respuesta cerrada sobre acciones unte una crisis epiléptica, global y por grupo

Total			Población general			Familiares			OR (IC95%)
Sí	No	NS	Sí	No	NS	Sí	No	NS	
*Permanecer junto a la persona hasta que termine la CE*									p=0,013
419 (96,5)	2 (0,5)	13 (3,0)	250 (94,7)	2 (0,8)	12 (4,5)	169 (99,4)	0	1 (0,6)	8,7 (1,1-67)
*Registrar la duración de la CE*									p=0,001
283 (65,2)	50 (11,5)	101 (23,3)	131 (49,6)	42 (15,9)	91 (34,5)	152 (89,4)	8 (4,7)	10 (5,9)	8,5 (4,9-14)
*Limpiar secreciones bucales/vómito*									p=0,006
253 (58,3)	100 (23)	81 (18,7)	140 (53)	56 (21,2)	68 (25,8)	113 (66,5)	44 (25,9)	13 (7,6)	1,7 (1,1-2,6)
*Abrir la boca con algún objeto*									p=0,001
186 (42,9)	181 (41,7)	67 (15,4)	131 (49,6)	77 (29,2)	56 (21,2)	55 (32,4)	104 (61,2)	11 (6,5)	0,3 (0,2-0,4)
*Aflojar la ropa alrededor del cuello*									p=0,592
374 (86,2)	24 (5,5)	36 (8,3)	225 (85,2)	13 (4,9)	26 (9,8)	149 (87,6)	11 (6,5)	10 (5,9)	1,2 (0,7-2)
*Sujetar a la persona durante la CE*									p=0,001
161 (37,1)	211 (48,6)	62 (14,3)	112 (42,4)	98 (37,1)	54 (20,5)	49 (28,8)	113 (66,5)	8 (4,7)	0,3 (0,2-0,5)

NS: no sé; IC: intervalo de confianza; CE: crisis epiléptica.

El resultado de la pregunta sobre la posición a colocar al paciente durante la CE fue similar en ambos grupos (p = 0,244). La respuesta correcta (decúbito lateral) predominó en ambos grupos con frecuencias similares, y también fueron muy similares las frecuencias de la respuesta de mantener a la persona en la posición en que está. Sin embargo, la frecuencia de la respuesta “boca arriba” casi se duplicó en el grupo de población general, mientras que en el grupo de familiares nadie respondió “boca abajo” (Tabla 3). Los conocimientos sobre primeros auxilios frente a una CE fueron adquiridos de distinto modo según el grupo (p<0,0001): el personal sanitario fue la fuente para casi la mitad de familiares y para el 2,4% de población general, mientras que medios de comunicación y redes sociales lo fueron para menos del 10% de familiares y para casi una cuarta parte de la población general (Tabla 3).

**Tabla 3:** Conocimientos sobre colocación de la persona con crisis epiléptica y fuente de los mismos según grupo

	Población general	Familiares
	(%)	(%)
*Posición a colocar al paciente*		
En la que está	8,3	9,4
Boca arriba	10,2	5,9
De lado	80,3	84,7
Boca abajo	1,1	0
*Método de aprendizaje*		
Cursos/charlas	31,5	12
Medios de comunicación y RRSS	23,4	9,8
Personal sanitario	2,4	44,6
Otro	42,8	33,7

RRSS: redes sociales.

En cuanto a los resultados del apartado de preguntas dirigidas a familiares de pacientes con epilepsia, casi la mitad de las personas encuestadas (48,9%) administraría alguna medicación al paciente, cuya prescripción fue realizada por especialista en Neurología en todos los casos, siendo la más frecuente midazolam por vía oral. Al presenciar una CE, casi un tercio llamaría siempre a emergencias, mientras la mayoría (63%) llamarían al 061 solo en determinadas situaciones (Tabla 4), siendo la duración prolongada de la CE el motivo más referido (41,3%). El 71% avisarían a los servicios de emergencias tras 5 o más minutos de CE. La mitad de los familiares (48,9%) pertenece a alguna asociación de epilepsia.

**Tabla 4:** Respuestas a preguntas dirigidas a familiares de pacientes con epilepsia

Respuesta	%
*Administrar medicación durante la CE*	
Sí	48,9
Midazolam oral	63,6
Diazepam rectal	15,9
Diazepam oral	9,1
Lorazepam oral	4,5
Levetiracetam oral	2,3
No	51,1
No sé	0
*Llamar al 112 / 061 durante la CE*	
Sí, siempre	32,6
En ciertas situaciones	63,0
Duración prolongada	41,3
Bajo nivel de consciencia	14,7
Traumatismo	12,1
Dificultad respiratoria	12,1
Crisis de repetición	12,1
Crisis diferente	4,9
Primera crisis	2,8
No, nunca	4,4
No sé	0
*Llamar al 112 / 061 si la CE dura más de*	
1 minuto	4,1
3 minutos	24,2
5 minutos	54,5
10 minutos	17,2

CE: crisis epiléptica.

## DISCUSIÓN

En el presente estudio se ha evaluado el conocimiento que la población general y los familiares de pacientes con epilepsia poseen acerca de la atención básica de primeros auxilios ante una CE. Siendo poblaciones demográficamente comparables, se observa una tendencia global a mayor proporción de respuestas adecuadas en el grupo de familiares destacando que, tanto en la pregunta abierta como en la cerrada de opción múltiple, el grupo de población general contestó de forma significativamente más frecuente que realizaría una acción inadecuada (como la introducción de un objeto en la cavidad oral de la persona que sufre la CE). En términos generales, se ha observado que el conocimiento que tienen sobre el tema no es óptimo y que existe un significativo margen de mejora.

Los resultados obtenidos están en consonancia con los hallados en otros trabajos científicos previos en distintas poblaciones^6^^,^^7^^,^^10^^,^^11^. En nuestro país, el Libro Blanco de la Epilepsia publicado en 2013, valoró el conocimiento y la percepción de esta enfermedad en distintas poblaciones y colectivos; uno de sus puntos se refería a las medidas de primeros auxilios ante una CE^20^. Aunque los resultados no son directamente comparables con nuestro trabajo ante las distintas poblaciones interrogadas y las diferencias en la propia encuesta, ya se detectó en ese momento la existencia de errores importantes y extendidos en relación con dicha atención.

En nuestro estudio, la pregunta abierta inicial nos enmarca el conocimiento subóptimo en este campo, ya que colocar al paciente en posición lateral de seguridad es la única acción adecuada que realizaría algo más de la mitad del grupo de familiares; todas las demás acciones las indican menos de la mitad de los encuestados de ambos grupos. También se observa que las acciones adecuadas serían realizadas en mayor medida por el grupo de familiares, resultado que concuerda con un estudio previo^11^, siendo lógica esta tendencia más proactiva dada la probable mayor experiencia, formación e interés por la epilepsia en esta población por su contexto familiar. Sin embargo, probablemente no se llega a un nivel óptimo de conocimiento teniendo en cuenta la mayor probabilidad de presenciar una CE que presenta este grupo.

Como dato positivo, hay que destacar el elevado porcentaje de sujetos que colocarían al paciente en decúbito lateral según la pregunta cerrada correspondiente (82 % en total) ya que los estudios realizados por Kanjo y col^8^ y Kangevari y col^11^ presentaron peores resultados al respecto (64,8% y 43,7% respectivamente). Además, prácticamente la totalidad de individuos permanecería con el paciente durante la CE, llegando al 100% en el grupo de familiares. Respecto a las preguntas específicas para este grupo, más de la mitad de los sujetos respondieron apropiadamente a la relativa a cuándo llamar al 112 / 061, indicando cinco minutos. La mayoría de ellos llamaría a emergencias solo en determinadas situaciones, exponiendo escenarios como la duración prolongada de la CE o el mantenimiento de un bajo nivel de consciencia.

Sin embargo, existen maniobras altamente peligrosas, como introducir objetos en la boca del paciente o sujetarlo durante la CE, que serían llevadas a cabo por un gran número de encuestados. Casi la mitad de los encuestados de la población general introduciría algún objeto en la boca y un 21,2% no saben si deberían realizar dicha maniobra, frente al 32,4% de familiares que lo haría. Aunque los porcentajes en familiares son menores comparados a la población general, siguen siendo preocupantes. La creencia errónea de introducir un objeto en la cavidad oral del paciente o incluso intentar sacar la lengua para evitar el ahogamiento es una de las más extendidas en la sociedad. Este concepto apareció en la literatura hacia mitad del siglo XIX; sin embargo, en el siglo XX los profesionales sanitarios ya no recomendaban este tipo de acción^15^. No obstante, esta idea persiste en la actualidad como numerosos estudios, incluyendo el presente, demuestran^6^^,^^8^^-^^11^. Incluso entre personal sanitario, hasta el 54% llevaría a cabo esta maniobra según el trabajo realizado por Martino y col^9^. Este hecho puede deberse a la relación que el profesional sanitario podría establecer con la protección bucal utilizada en la terapia electroconvulsiva. Sin embargo, estas protecciones bucales son adecuadas y se colocan apropiadamente antes del tratamiento, algo imposible en el escenario de una CE, estando por lo tanto contraindicada cualquier maniobra que intente emular este manejo^15^.

La persistencia de estas creencias erróneas ha podido suceder en parte gracias a las ficciones narrativas escritas y audiovisuales, y a los medios de comunicación. En el presente trabajo, hasta un 23,4% de la población general afirma adquirir estos conocimientos gracias a los medios de comunicación. Otro estudio informó un porcentaje del 34,9% de conocimiento en la materia a través de internet y redes sociales^7^, medios que pueden ofrecer una oportunidad formativa pero en los cuales no existe un control riguroso sobre la fiabilidad del contenido científico. Existen numerosos ejemplos de películas y obras narrativas en las que se representan CE y se observan acciones no adecuadas en su auxilio, incluso en obras audiovisuales de ficción médica^21^^,^^22^. Además, cabe destacar que, aunque existen diversos tipos de CE, el mundo del arte parece tener una predilección por reproducir las crisis tónico-clónicas generalizadas respecto a otros tipos, probablemente por su presentación más espectacular adecuada para fines dramáticos^21^, y en ocasiones representadas en relación con impulsos violentos y e incluso con crímenes. Esto nos permite observar no solo el desconocimiento que hay sobre el manejo de las CE sino también el posible estigma y prejuicio subyacente que aún existe en relación con la epilepsia y las CE en sí mismas.

Todos estos datos deben hacernos reflexionar sobre la importancia de mejorar el conocimiento y la formación de la población no sanitaria en primeros auxilios en epilepsia. Una revisión reciente ha observado que los programas formativos sobre esta materia, enfocados a diferentes colectivos (población general, niños en etapa escolar, profesores...), mejoran las actitudes frente a las CE, optimizando su atención y disminuyendo la realización de maniobras peligrosas. En este mismo trabajo se sugieren vías alternativas para la realización y difusión de dichas intervenciones educativas y entrenamiento sobre primeros auxilios, sugiriendo el uso de las diferentes redes sociales como forma de hacer llegar dicha información principalmente a la población más jóven^23^. Sin embargo, a pesar de la oportunidad que estas plataformas podrían brindar para la difusión de estos contenidos, sería deseable desarrollar estrategias para asegurar que los mismos cumplen unos estándares de calidad científica para evitar ahondar en actitudes e información errónea como las referidas previamente, hecho que ya se ha estudiado en una red social^24^. Diferentes intervenciones educativas han demostrado aumentar el conocimiento, actitudes positivas y aproximación a los primeros auxilios en epilepsia: mediante cursos presenciales en el contexto de programas formativos escolares^25^, a través de sesiones que incluyen dinámicas de *role playing*^26^, o mediante cursos libres *online* certificados^27^.

Entre las limitaciones del presente estudio se encuentra el propio modelo de obtención de información a través de una encuesta, con la posibilidad de sesgo de repuesta que conlleva. Para intentar limitarlo se introdujo la pregunta abierta inicial. Si bien esta pregunta presenta también cierta limitación ya que puede infravalorar los conocimientos que los individuos poseen, evita el sesgo que se puede producir con las preguntas de opción múltiple. Probablemente este enfoque pueda acercarnos al nivel real de conocimientos de una forma más adecuada que solo mediante una encuesta con preguntas cerradas, lo que es una fortaleza y un dato diferenciador respecto a otros estudios de temática similar. Otra limitación es un posible sesgo de selección del grupo de familiares al provenir únicamente de dos centros hospitalarios, lo que puede influir hasta cierto punto en su validez externa. Por último, un aspecto positivo es poder evaluar y comparar los resultados entre una muestra de población general y familiares de pacientes con epilepsia, ya que pocos trabajos previos analizan a este último grupo^28^^,^^29^, especialmente en nuestro entorno.

En conclusión, los resultados del presente estudio sugieren que el nivel de conocimiento de la población general y de familiares de pacientes con epilepsia sobre primeros auxilios ante una CE no es óptimo y que se necesita una mayor formación al respecto.

Se sugieren distintas líneas de actuación: 1) Respecto a la práctica clínica habitual, estos datos nos deben llevar a plantear reservar cierto tiempo de las consultas -tanto de atención primaria como de neurología y epilepsia- para tratar el tema de los conocimientos sobre CE. Sería positivo implementar consultas específicas de primera CE y consultas de enfermería de epilepsia. 2) Respecto a la población general, algunas medidas para mejorar su actitud y aptitud podrían ser: organizar cursos de formación presenciales a través de los centros de atención primaria y en coordinación con servicios de neurología; difundir programas o artículos informativos a través de medios de comunicación clásicos (televisión, prensa escrita); difundir sesiones e información con el aval de sociedades científicas mediante internet y redes sociales, que dada la inmediatez propia de estos nuevos medios de comunicación, tendrían una especial utilidad en la difusión de mensajes sencillos, claros y rápidos (por ejemplo, colocación del paciente, o no introducir objetos en la boca) a una gran cantidad de población. 3) Es importante fomentar la educación sanitaria general desde edades tempranas, por lo que asegurar y reforzar la inclusión de estos temas en los planes de estudios escolares puede sentar las bases para mejorar el conocimiento global y actitudes ante la epilepsia. La eficacia de estas intervenciones en la adquisición de conocimiento y aptitudes podría ser evaluada en futuros estudios. Todo ello puede ayudar a mejorar la atención más básica, pero de vital importancia, de los pacientes con epilepsia.

## Data Availability

Se encuentran disponibles bajo petición al autor de correspondencia.

## ANEXO

### Anexo Encuesta sobre conocimiento de primeros auxilios ante una crisis epiléptica/convulsiones

Nos dirigimos a usted para invitarle a participar en un trabajo de investigación médica mediante una **encuesta anónima** sobre el conocimiento de las medidas básicas de primeros auxilios ante una crisis epiléptica. No le llevará más de 10 minutos de su tiempo contestarla. Le agradecemos de antemano su participación.

Datos demográficos

1. Edad _______________

2. Sexo

□ Mujer □ Hombre

3. ¿Es usted familiar cercano o convive con un paciente con epilepsia?

□ Sí | □ No

4. Lugar de residencia _______________

5. Nivel de estudios

□ Primaria, Secundaria, Bachiller, Formacion profesional (FP) grado medio

□ FP grado superior, Carrera universitaria, Master, doctorado

Conocimiento sobre primeros auxilios ante una crisis epiléptica

1. ¿Cómo actuaría y qué acciones realizaría si presenciara que alguien sufre una crisis epiléptica/convulsiones? Responda brevemente con sus propias palabras __________________________________________

2. ¿Permanecería con la persona que está sufriendo convulsiones hasta que la crisis terminara?

□ Sí | □ No | □ No lo sé

3. ¿Intentaría registrar el tiempo que duran las convulsiones?

□ Sí | □ No | □ No lo sé

4. ¿En qué posición colocaría a la persona que está sufriendo las convulsiones?

□ La mantendría en la posición en la que está

□ La acostaría boca arriba

□ La acostaría de lado

□ La acostaría boca abajo

5. ¿Intentaría limpiar secreciones bucales o vómito si la persona que está sufriendo las convulsiones las tuviera?

□ Sí | □ No | □ No lo sé

6. ¿Pondría algún objeto para abrir la boca de la persona que está sufriendo las convulsiones?

□ Sí | □ No | □ No lo sé

7. ¿Intentaría aflojar ropa ajustada que la persona que está sufriendo las convulsiones pudiera llevar alrededor del cuello?

□ Sí | □ No | □ No lo sé

8. ¿Intentaría sujetar a la persona que está sufriendo las convulsiones?

□ Sí | □ No | □ No lo sé

9. ¿Cómo ha aprendido lo que sabe sobre este tema?

□ Formación: colegio, instituto, charlas...

□ Medios de comunicación: televisión, radio, internet, redes sociales...

□ Explicaciones por parte del médico, enfermera...

□ Otro

SOLO CONTESTAR SI ES USTED FAMILIAR DE UN PACIENTE CON EPILEPSIA

(preguntas de 10 a 16)

10. ¿Administraría algún medicamento de rescate si su familiar estuviera sufriendo una crisis epiléptica?

□ Sí | □ No | □ No lo sé

11. **Solo** contestar **si en la anterior pregunta ha marcado SÍ**: ¿Qué medicación de rescate administraría? (nombre de fármaco y vía de administración). __________________________________________

12. **Solo contestar si en la pregunta 11 ha marcado SÍ**: Dicho medicamento, ¿por quién ha sido prescrito?

□ Neurólogo | □ Médico de familia | □ No prescripción médica | □ Otros

13. ¿Llamaría usted al 112/061 si su familiar estuviera sufriendo una crisis epiléptica?

□ Sí, siempre | □ Sólo en ciertas situaciones | □ No | □ No lo sé

14. **Solo contestar si en la pregunta 13 ha marcado SOLO EN CIERTAS SITUACIONES**: ¿A partir de cuánto tiempo de duración de crisis epiléptica llamaría usted al 112/061?

□ 1 minuto | □ 3 minutos | □ 5 minutos | □ 10 minutos

15. **Solo contestar si en la pregunta 13 ha marcado SOLO EN CIERTAS SITUACIONES**: ¿En qué situaciones llamaría usted al 112/061 si su familiar estuviera sufriendo una crisis epiléptica? Responda brevemente. __________________________________________

16. ¿Pertenece a alguna asociación de pacientes o familiares de pacientes con epilepsia?

□ Sí | □ No
